# Synthesis, Structure, and Spectroscopy of the Biscarboranyl Stannylenes (**bc**)Sn·THF and K_2_[(**bc**)Sn]_2_ (**bc** = 1,1′(*ortho*-Biscarborane)) and Dibiscarboranyl Ethene (**bc**)CH=CH(**bc**)

**DOI:** 10.1021/acs.organomet.3c00190

**Published:** 2023-06-26

**Authors:** Alice C. Phung, James C. Fettinger, Philip P. Power

**Affiliations:** Department of Chemistry, University of California, 1 Shields Avenue, Davis, California 95616, United States

## Abstract

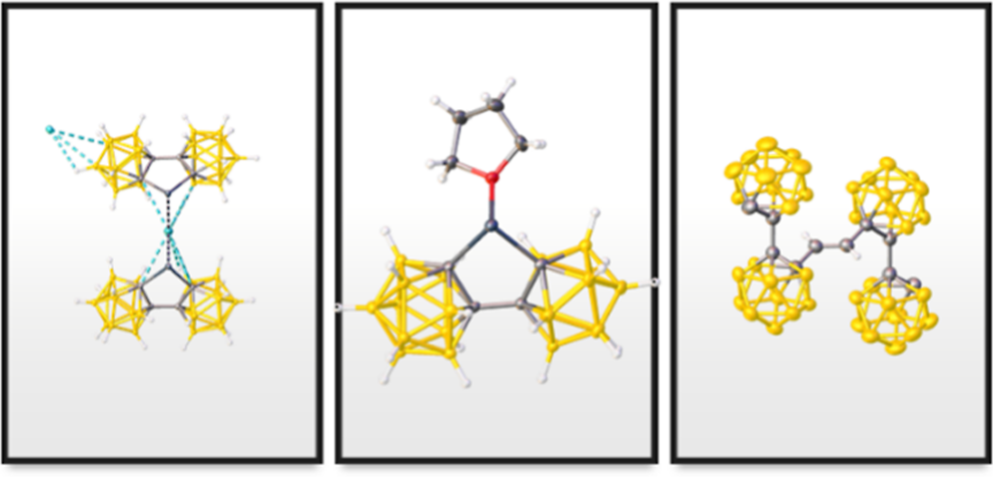

Two compounds containing a Sn(II) atom supported by a bidentate biscarborane ligand have been synthesized via salt metathesis. The synthetic procedures for (**bc**)Sn·THF (**bc** = 1,1′ (*ortho*-carborane) (**1**) and K_2_[(**bc**)Sn]_2_ (**2**) involved the reaction of K_2_[**bc**] with SnCl_2_ in either a THF solution (**1**) or in a benzene/dichloromethane solvent mixture (**2**). Using the same solvent conditions as those used for **2** but using a shorter reaction time gave a dibiscarboranyl ethene (**3**). The products were characterized by ^1^H, ^13^C, ^11^B, ^119^Sn NMR, UV–vis, and IR spectroscopy, and by X-ray crystallography. The diffraction data for **1** and **2** show that the Sn atom has a trigonal pyramid environment and is constrained by the **bc** ligand in a planar five-membered C_4_Sn heterocycle. The ^119^Sn NMR spectrum of **1** displays a triplet of triplets pattern signal, which is unexpected given the absence of a Sn–H signal in the ^1^H NMR, IR spectrum, and X-ray crystallographic data. However, a comparison with other organotin compounds featuring a Sn atom bonded to carboranes reveal similar multiplets in their ^119^Sn NMR spectra, likely arising from long-range nuclear spin–spin coupling between the carboranyl ^11^B and ^119^Sn nuclei. Compound **3** displays structural and spectroscopic characteristics typical of conjugated alkenes.

## Introduction

The charge-neutral compound 1,1′-bis(*ortho*-carborane) (**H**_**2**_**-bc**), often described as a three-dimensional aromatic analogue of biphenyl, is an interesting ligand for the support of stannylenes due to its steric bulk and strong κ^2^-binding that can form strained five-membered metallacycles.^1−3^ The majority of **bc** ligand metal complexes feature a transition metal that is κ^2^-C,C- or κ^2^-B,C-bonded to the **bc** ligand and stabilized by an aryl or alkyl group^3−11^ or another **bc** ligand.^3,12,13^ In these cases, the central transition metal is constrained to a square planar or tetrahedral geometry due to the rigid nature of the **bc** ligand scaffold. Additionally, there are reports of deboronated **bc**-based transition-metal complexes incorporating the transition-metal atom into the **bc** cage.^3,14−16^

**Figure 1:**
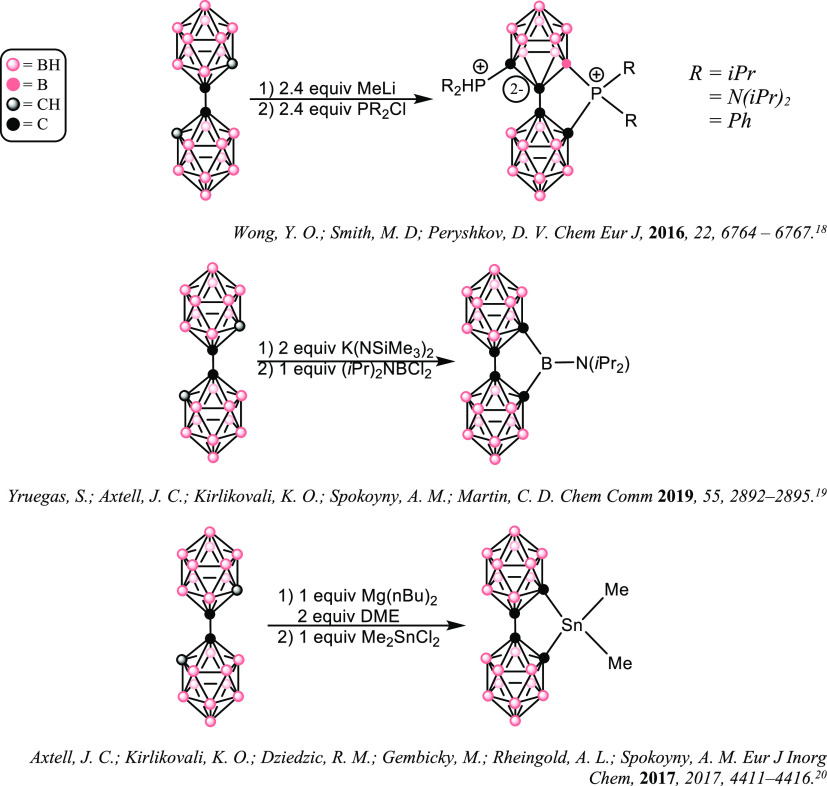
Synthetic routes of other **bc**-supported main-group metal complexes.

In contrast, there are relatively few main group metal complexes stabilized by a **bc** ligand,^17−22^ and the synthesis of these complexes has required activation of the C–H vertices of **H**_**2**_**-bc**. Since the boron-bonded hydrogens are hydridic while the carbon-bonded hydrogens are protic,^1,2^ lithiation is a common route for the C–H activation of **H**_**2**_**-bc**. The phosphorus complex *closo*-(C_2_B_10_H_10_)(PR_2_)-*nido*-(C_2_B_10_H_9_) (PHR_2_) (R = *i*Pr, N(*i*Pr)_2_, or Ph) describes the activation of **H**_**2**_**-bc** by lithiation to produce the dilithio salt.^18^ Alternatively, the synthesis for the 9-borafluorene three-dimensional analogue (**bc**)B(N(*i*Pr)_2_) generates the dipotassium salt of **bc** via potassium bis(trimethylsilyl)amide prior to a salt metathesis reaction with (*i*Pr_2_)NBCl_2_.^19^ Currently, the only known **bc** complex containing a heavy group 14 metal is the Sn(IV) complex, (**bc**)SnMe_2_, synthesized via reaction of the Grignard intermediate (**bc**)Mg(DME)_2_ (DME = 1,2-dimethoxyethane) with SnMe_2_Cl_2_ (Figure 1).^20^

Unlike the rarity of bis-carboranyl group 14 complexes, several *ortho*- and *meta*-carboranes containing B–Sn and C–Sn bonds are known.^23,24^ The earliest reports in 1965 concerned the trialkylcarboranyl tin complexes (C_2_B_10_H_10_)(SnR_3_)_2_, (R = alkyl), with each carbon vertex of the carborane cage bonded to a Sn(IV) atom, although structural data was not provided.^25^ The first isolable carboranyl tin structures were the organotin complexes [*o*-C_2_B_10_H_10_(CH_2_NMe_2_)SnR_2_Br (R = Me or Ph; X = Cl or Br) which feature a Sn(IV) bonded to a carbon vertex and stabilized by a Lewis basic −CH_2_NMe_2_ chelating group (Table 2 and ref (26)).^26^ In general, the majority of the tin-carborane complexes are achieved through an initial lithiation step in the stannylation of the C–H vertices of the carboranes cages.^16,23−38^

**Scheme 1:**
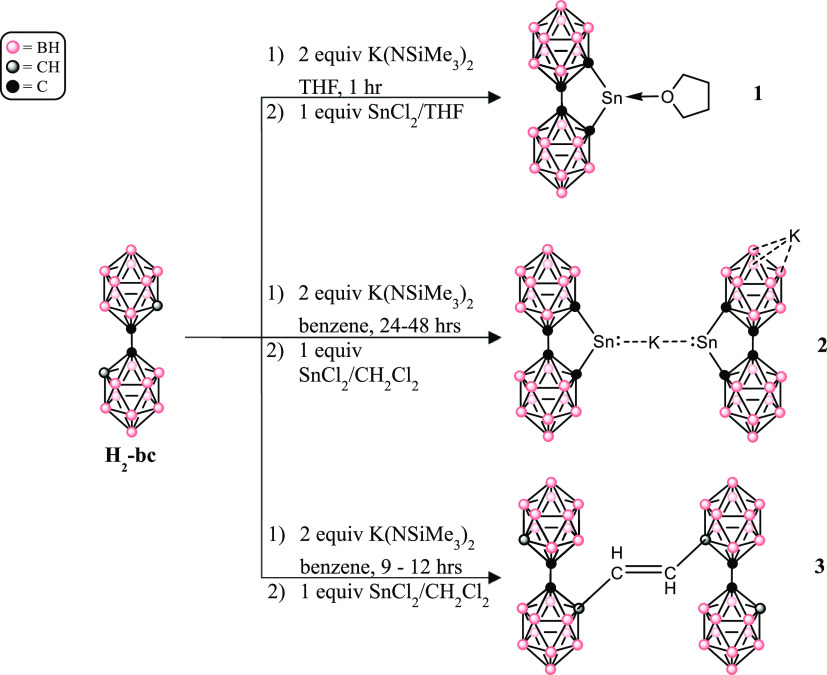
Syntheses of **1–3**

Monomeric, homoleptic stannylenes of the formula SnR_2_ are usually supported by bulky organic or related ligands such as alkyl, aryl, silyl, amido, alkoxo, thiolato, etc.^39−41^ Given the bulkiness and rigidity of **H**_**2**_**-bc**, the compound may be a suitable platform to support a stannylene, as biscarborane-supported stannylenes are not known prior to this work. Herein, we present the synthesis and characterization of complexes containing a 1,1′-bis(*o*-carboranyl) stannylene (**bc**)Sn moiety. These compounds were obtained by first deprotonating **H**_**2**_**-bc** via potassium bis(trimethylsilyl)amide (KHMDS) to create the potassium salt, K_2_[**bc**],^9^ which was then added to SnCl_2_ in THF. The reaction of K_2_[**bc**] with SnCl_2_ in a THF solution gives the THF-coordinated (**bc**)Sn·THF (**1**), while a benzene/dichloromethane mixture affords K_2_[(**bc**)Sn]_2_ (**2**). Shortening the reaction tme of the dipotassium salt from 24 h to 9 h prior to addition to a dichloromethane solution of SnCl_2_ produced the alkene (**bc**)CH=CH(**bc**) (**3**) (Scheme 1), presumably through a coupling reaction between the mono-deprotonated K[**H-bc**] salt and CH_2_Cl_2_ solvent molecules. X-ray crystallography and ^1^H NMR, ^11^B NMR, ^13^C NMR, and UV–vis spectroscopy show that the (**bc**)Sn moiety in complexes **1** and **2** confer structural and spectroscopic similarities between the two. Compound **1** was further characterized by ^119^Sn NMR spectroscopy. Characterization by X-ray crystallography, ^1^H, ^11^B, ^13^C NMR, UV–vis, and IR spectroscopy of compound **3** confirms its conjugated alkene structure.

## Experimental Section

### General Procedures

All manipulations were carried out by using modified Schlenk techniques under a N_2_ atmosphere. Solvents were dried over columns of activated alumina using a Grubbs-type purification system (Glass Contour), stored over Na (THF, toluene) mirrors, K (diethyl ether, hexanes) mirrors, or 3 Å molecular sieves (dichloromethane) and degassed via three freeze–pump–thaw cycles prior to use. KHMDS was purchased from Sigma-Aldrich and washed three times with hexanes prior to use. The compound **H**_**2**_**-bc** was synthesized according to literature procedures.^10,42^ The ^1^H, ^11^B{1H}, ^13^C{^1^H}, and ^119^Sn{^1^H} NMR spectra were recorded on a Bruker AVANCE DRX 500 MHz spectrometer and the ^1^H and ^13^C{^1^H} spectra were referenced to the residual solvent signals in C_6_D_6_ (^1^H: δ 7.15 ppm, ^13^C: δ 128.06 ppm).^43^ UV–visible spectra were recorded using dilute hexane solutions in 3.5 mL quartz cuvettes using an Olis 17 Modernized Cary 14 UV–vis/NIR spectrophotometer. Infrared spectra for **1** and **2** were recorded as Nujol mulls between CsI windows on a PerkinElmer 1430 spectrophotometer. The infrared spectrum for **3** was collected on a Bruker Tensor 27 ATRFTIR spectrometer. Melting points were determined on a Meltemp II apparatus in flame-sealed glass capillaries equipped with a partial immersion thermometer.

#### (**bc**)SnTHF (**1**)

THF (ca. 50 mL) was added to a flask containing **H**_**2**_**-bc** (0.50 g, 1.75 mmol) and KHMDS (0.69 g, 3.5 mmol) and stirred at room temperature for 1 h. The resulting K_2_[**bc**] solution was then added to a room-temperature THF suspension of SnCl_2_ (0.33 g, 1.75 mmol). The solution was stirred overnight to afford a pale pink solution. The THF was removed under reduced pressure, and the resulting dark pink solid was re-dissolved in ca. 40 mL of warm toluene. Filtration through a Celite plug gave a pale-yellow solution. The toluene was removed under reduced pressure, and the solid was re-dissolved in dichloromethane. Concentration of the dichloromethane solution to ca. 10 mL and storage at ca. −18 °C gave pale yellow crystals of **1**. Yield: 0.57 g (70%). mp 250–260 °C. ^1^H NMR (500 MHz, C_6_D_6_, 20 °C): δ 1.40 (m, 4H, THF C*H*_2_(3,4)) δ 1.41–3.40 (m, B*H*), and δ 3.55 (m, 4H, THF C*H*_2_(2,5)). ^11^B{^1^H} NMR (160.5 MHz, C_6_D_6_, 20 °C) δ −11.47 (5B), δ −9.33 (6B), δ −8.12 (5B), δ 1.27 (2B), and δ 0.59 (2B). ^13^C{^1^H} NMR (151 MHz, C_6_D_6_, 20 °C): δ 24.95 (THF *C*H_2_(3,4), δ 62.91 (**bc***C*) δ 69.99 (THF *C*H_2_(2,5)), and δ 71.81 (**bc***C*). ^119^Sn NMR (149 MHz, C_6_D_6_, 20 °C): δ −137.31 (^2^*J*_119Sn_–_11B_ = 1487 Hz). UV–Vis (toluene): λ_max_ (ε) 280 nm (15,000 mol^–1^ L cm^–1^) 345 nm (9600 mol^–1^ L cm^–1^).

#### K_2_[(**bc**)Sn]_2_ (**2**)

Benzene (ca. 50 mL) was added to a flask containing **H**_**2**_**-bc** (0.50 g, 1.75 mmol) and KHMDS (0.69 g, 3.5 mmol) and stirred at room temperature until a tan-colored solution was achieved (approx. 24–48 h). The K_2_[**bc**] solution was then added directly to a room-temperature dichloromethane solution of SnCl_2_ (0.33 g, 1.75 mmol). The solution was stirred overnight to yield a pale pink solution. The solvent was removed under reduced pressure, and the orange solid was re-dissolved in warm toluene and separated from the light gray solid by filtration. Toluene was removed under reduced pressure, and the solid was re-dissolved in dichloromethane. Pale yellow crystals of **2** were grown from a concentrated dichloromethane solution (ca. 10 mL) stored at room temperature overnight. Yield: 0.39 g (50%). mp 240 °C. ^1^H NMR (600 MHz, C_6_D_6_, 20 °C): δ 1.50–3.50 (m, B*H*). ^11^B{^1^H} NMR (160.5 MHz, C_6_D_6_, 20 °C) δ −11.47 (5B) δ −9.33 (6B), δ −8.12 (5B), δ 1.27 (2B), and δ 0.59 (2B). ^13^C{^1^H} NMR (151 MHz, C_6_D_6_, 20 °C): δ 62.91 (**bc***C*), and δ 71.62 (**bc** C). ^119^Sn NMR signal not observed. UV–vis (toluene): λ_max_ (ε) 280 nm (3700 mol^–1^ L cm^–1^) 345 nm (820 mol^–1^ L cm^–1^).

#### (**bc**)_2_(CH)_2_ (**3**)

Benzene (ca. 50 mL) was added to a flask containing **H**_**2**_**-bc** (0.50 g, 1.75 mmol) and KHMDS (0.69 g, 3.5 mmol) and stirred at room temperature for 9–12 h. The pale-yellow slurry was then added directly to a room-temperature dichloromethane solution of SnCl_2_ (0.33 g, 1.75 mmol). The solution was stirred overnight until all SnCl_2_ solids were solubilized, affording a pale yellow-orange solution. The solvent was removed under reduced pressure, and the orange solid was re-dissolved in warm toluene to filter off the white solid. Toluene was removed under reduced pressure, and the product was re-dissolved in ca. 10 mL of benzene. Concentration of the benzene solution of the product to ca. 1 mL and storage overnight at room temperature gave yellow-orange crystals of **3**. Yield: 0.27 g (50%). mp 260–270 °C. ^1^H NMR (500 MHz, C_6_D_6_, 20 °C): δ 1.40–3.50 (m, B*H*), δ 3.78 (s, 2H, cage C*H*), δ 5.44 (s, 1H, olefinic C*H*), and δ 6.10 (s, 1H, C=C*H*). ^11^B{^1^H} NMR (160.5 MHz, C_6_D_6_, 20 °C) δ −11.47 (8B), δ −9.33 (11B), δ −8.04 (9B), δ −6.37 (2B), δ 1.27 (5B), and δ 0.56 (5B). ^13^C{^1^H} NMR (151 MHz, C_6_D_6_, 20 °C): δ 2.65 (olefinic *C*H), δ 62.91 (**bc***C*), and δ 71.82 (**bc** C). UV–vis (toluene): λ_max_ (ε) 284 nm (780 mol^–1^ L cm^–1^), 334 nm (290 mol^–1^ cm^–1^). AT-FTIR: ν_=CH_ 3063 (s), ν_=CH_ 1254.13 (s), ν_=CH_ 1069.56 (s), ν_=CH_ 716.55 (s).

## Results and Discussion

### Synthesis

C–H activation in organometallic species often involves their treatment with alkyl lithium reagents to create a reactive C–Li bond. Working with the biscarborane system presents an interesting synthetic challenge, as both the hydridic B–H and protic C–H vertices of **H**_**2**_**-bc** are potentially susceptible to lithiation,^44,45^ with the lack of selectivity previously noted to lead to isomers^10^ or cage-opened products.^17,18,46^ Peryshkov and co-workers in 2016 had intended to synthesize an “independently C-substituted biscarborane cluster” and bind a phosphorus atom to the **bc** ligand through the carbon vertices in κ^1^-mode.^18^ However, addition of a dialkylphosphine chloride to the Li_2_[**bc**]/THF solution gave an asymmetric scaffold, with one of the carborane cages of the **bc** molecule undergoing a cage-opening reaction to produce the *closo*-(C_2_B_10_H_10_)-*nido*-(C_2_B_10_H_9_) backbone.^18^*Nido*-carboranyl species are a known decomposition product of **H**_**2**_**-bc** in the presence of a strong base or nucleophile.^44,47−49^

Synthetic methods for selective **bc** vertex-activation were first reported in 2018 with the (**bc**)Pt(dtb-bpy) (dtb-bpy = 4,4′-di-*tert*-butyl-2,2′-bipyridine) isomers.^9^ The κ^2^-C,C-bound isomer was generated by reacting **H**_**2**_**-bc** with 2 equiv of the non-nucleophilic and mild base potassium bis(trimethylsilyl)amide (KHMDS) and the κ^2^-B,C-bound isomer was generated by reacting **H**_**2**_**-bc** stepwise with 1 equiv of KHMDS and 1 equiv of MeLi.^9^ This method of selectively activating the C–H vertices without forming deboronated *nido*-carboranyl side products via a non-nucleophilic, mild base was utilized to generate compounds **1–3**.

Initially, following the procedure of Spokoyny and coworkers^9^ produced a tan-colored THF solution of K_2_[**bc**] which was added to a THF suspension of 1 equiv of SnCl_2_ and resulted in the isolation of compound **1**. Recrystallization from dichloromethane gave pale yellow crystals of **1**. X-ray crystallographic data revealed a THF molecule bound to the central Sn atom in addition to the bc ligand.

The synthesis of **2** proceeded similarly to that of **1** but with the difference that the THF solvent was replaced with a benzene/dichloromethane mixture (Scheme 1). Generating K_2_[**bc**] in a benzene solution required increased time due to the low solubility of the dipotassium salt in benzene in comparison to that in THF. Once a benzene solution assumed the same tan color as the K_2_[**bc**]/THF solution, approx. 24–48 h at room temperature, addition to a rapidly stirring dichloromethane solution of 1 equiv of SnCl_2_ gave, after workup and recrystallization in the same manner as **1**, light orange crystals of **2**.

Compound **3** was synthesized by a procedure similar to that of **2**, with the only difference being the amount of time the benzene solution was allowed to stir (Scheme 1). Stirring 1 equiv of **H**_**2**_**-bc** with 2 equiv of KHMDS in benzene for approx. 9–12 h afforded an ivory-colored to pale-yellow solution which was then added to a rapidly stirring dichloromethane solution of 1 equiv of SnCl_2_. Workup and recrystallization from benzene gave pale-orange crystals of **3**. The additional carbon atoms to afford the C=C bridging fragment are from the dichloromethane solvent. Given the pale color of the K_2_[**bc**] benzene solution observed with the shortened reaction time, it is likely that the KHMDS had activated only one C–H vertex prior to addition to the SnCl_2_/CH_2_Cl_2_ solution. This mono-activated K[**H-bc**] proceeded to react with the solvent molecules to afford a C=C bond. The reaction was repeated without SnCl_2_, but compound **3** was not generated, suggesting that SnCl_2_ is required to create the bridging alkene, possibly via a coupling mechanism similar to the Stille reaction.^50^

### X-ray Crystal Structures

Due to the rigid nature of the **bc** ligand, the stannylenes in **1** and **2** are constrained to a five-membered C_4_Sn cycle. The sum of the angles of the stannocycles equal 533.45° in **1** and 538.55 and 538.96° in **2**, indicating an essentially planar C_4_Sn cyclic moiety. The C–C bond that links the carborane cages together in **1** and **2** is in the range 1.532(5)–1.542(4) Å, which is slightly shorter than the C–C bond in the **H**_**2**_**-bc** precursor (1.602(2)).^51^ Additionally, the Sn–C bonds of **1** and **2** are 2.272(3)–2.309(3) Å (Table 1), slightly longer than the sum of the covalent radii of Sn (1.40 Å) and C (0.75 Å).^52^ The shortened C_cage_–C_cage_ bond and the minor elongation of the Sn–C single bond likely function to relieve strain to accommodate the larger Sn atom into the planar heterocycle. This constrained framework has also forced a narrow sub-90° angle at the central Sn atom at 83.05(12)° in **1** and 81.69 and 81.86° in **2** (Table 1), enabling a C–Sn–C bond angle narrower than other 5-membered organotin heterocycles (82.9(9)–93.8(2)°).^53−64^

**Table 1:** Selected Structural Data for **1**–**3**

compound	**1**	**2**	**3**
C_cage_–Sn, Å	2.272(3), 2.279(4)	Sn1: 2.276(3), 2.309(3)	
		Sn2: 2.288(4), 2.289(3)	
Sn–O or Sn–K, Å	2.249(3)	Sn1: 2.5876(8)	
		Sn2: 2.5855(9)	
C_cage_–Sn–C_cage_, deg	83.05(12)	Sn1: 81.69(11)	
		Sn2: 81.86(12)	
C–Sn–THF or C–Sn–K, deg	90.95(12), 93.12(12)	Sn1: 88.23(10), 94.11(7)	
		Sn2: 92.68(7), 90.89(7)	
C=C, Å			1.319(4)
C_cage_–C_olefin_, Å			1.488(3)
C_cage_–C_olefin_–C_olefin_, deg			123.1(1)

**Figure 2:**
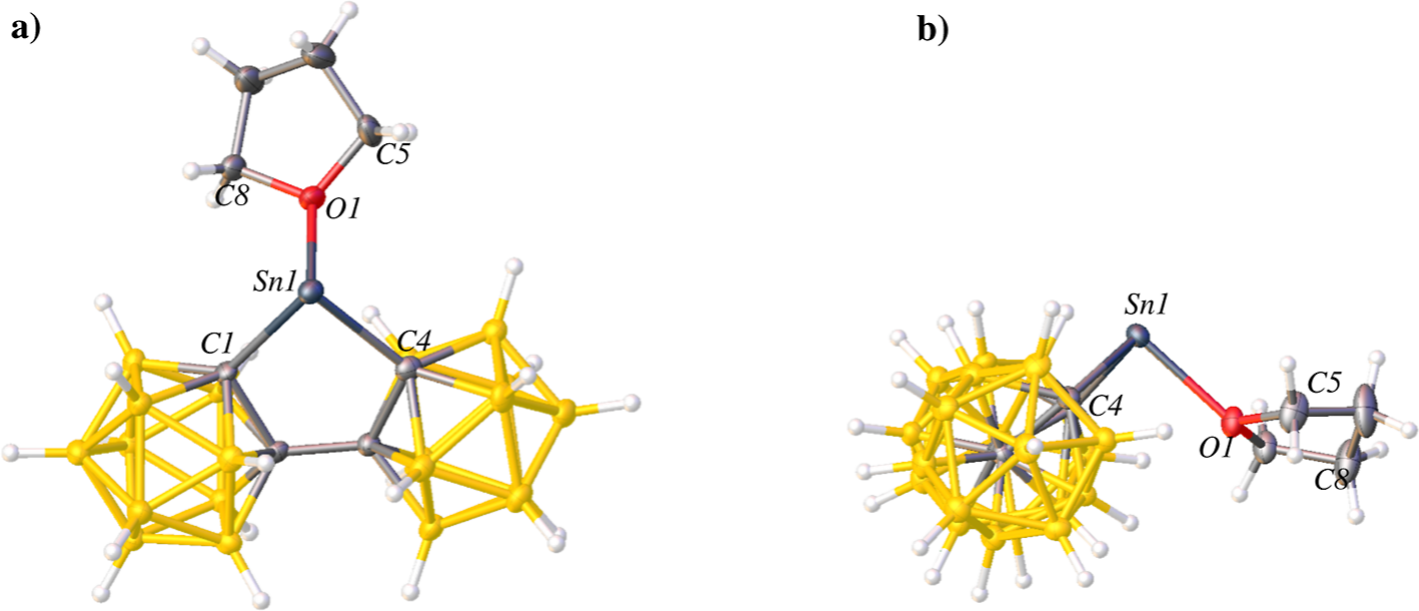
Thermal ellipsoid plot (50%) of **1**. CH_2_Cl_2_ solvent molecules are not shown for clarity. (a) “Top” view of **2**. (b) “Side” view of **2**. Selected bond lengths (Å) and angles (deg): C1–Sn1 = 2.272(3), C4–Sn1 = 2.279(4), O1–Sn1 = 2.249(3), C1–Sn1–C4 = 83.05(12), C1–Sn1–O1 = 90.95(12), C4–Sn1–O1 = 93.12(12).

Compound **1** co-crystallizes with two dichloromethane molecules and shows that a THF molecule is coordinated to the κ^2^-C,C-bonded Sn atom. The Sn–O_THF_ distance of 2.239(3) Å is within the range of other Sn–O_THF_ distances in THF-coordinated Sn(II) complexes (2.261(14)–2.422(6) Å),^65−68^ consistent with a dative Sn ← O interaction. Additionally, the THF molecule is bonded to the Sn atom at approximately perpendicular to the C_4_Sn plane, with C–Sn-O_THF_ angles at 90.95(12) and 93.12(12)° (Figure 2b). In total, the sum of the angles around the tin atom equals 267.12(12)° and indicates a highly pyramidalized geometry. The coordination geometry at Sn is typical of other THF-coordinated Sn complexes, which report C–Sn–O_THF_ angles in the range 84.8(3)–94.6(6)°.^65−68^

**Figure 3:**
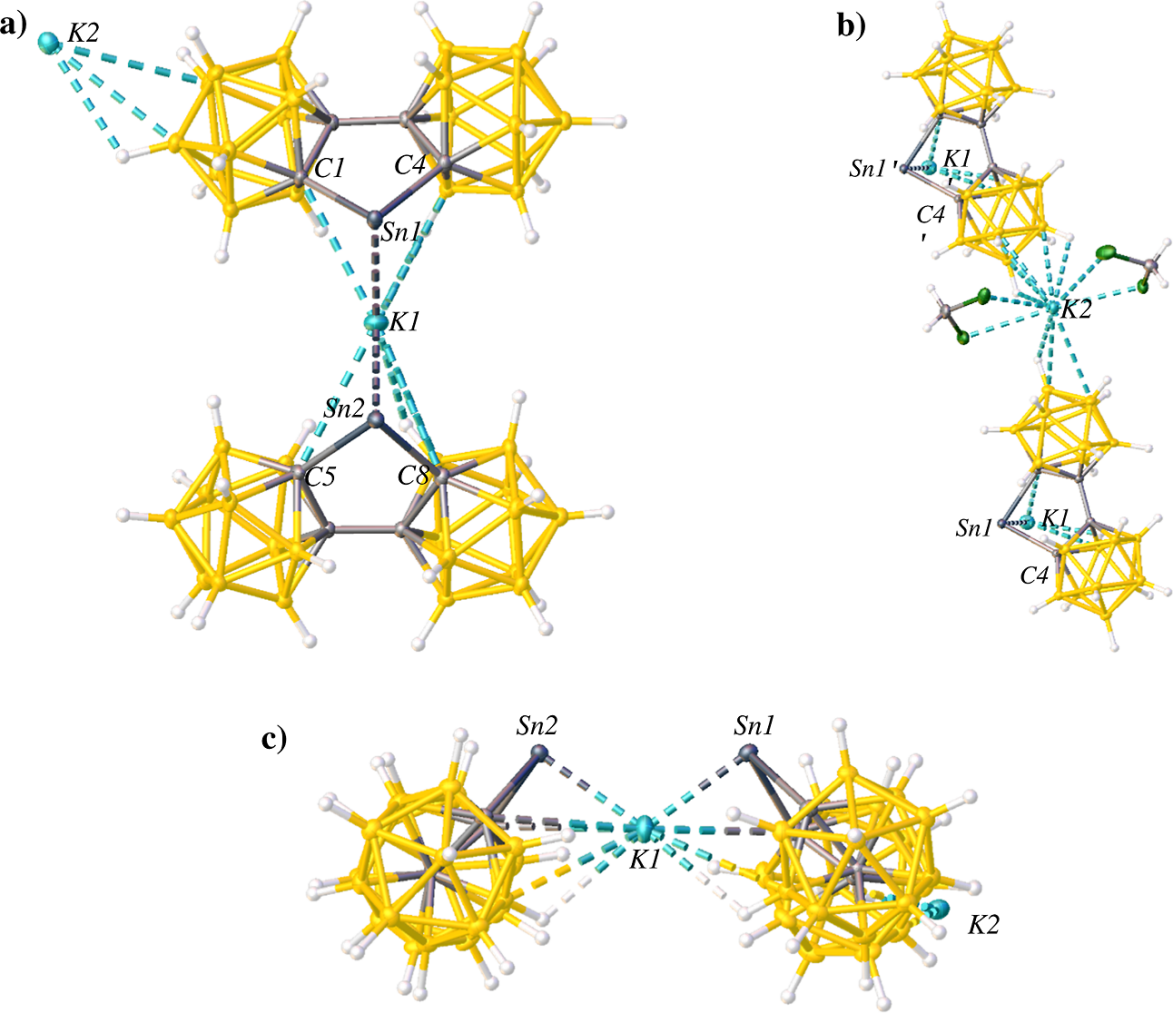
Thermal ellipsoid plot (50%) of **2**. (a) “Top” view of **2** to show coordination of K1. CH_2_Cl_2_ solvent molecules are not shown for clarity. (b) Expanded view of **2** to show coordination of K2. (c) “Side” view of **2** to show coordination of K1. CH_2_Cl_2_ solvent molecules are not shown for clarity. Selected bond lengths (Å) and angles (deg): C1–Sn1 = 2.276(3), C4–Sn1 = 2.309(3), C5–Sn2 = 2.288(4), C8–Sn2 = 2.289(3), K1–Sn1 = 2.5876(8), K1–Sn2 = 2.5855(9), C1–Sn1–C4 = 81.69(11), C5–Sn2–C8 = 81.86(12), C1–Sn1–K1 = 88.23(10), C4–Sn1–K1 = 94.11(7), C5–Sn2–K1 = 92.68(7), C8–Sn2–K1 = 90.89(7).

Compound **2** co-crystallizes with two dichloromethane molecules as well as two K^+^ ions from K_2_[**bc**] in the first step in the synthesis. One K^+^ ion forms a Sn–K–Sn bridging fragment between two (**bc**)Sn moieties (Figure 3a), and the other K^+^ ion appears as a counterion coordinated to the B–H vertices of the **bc** cage (Figure 3b). Compound **2** is unusual in that the Sn–K distances at 2.5866(8) and 2.5855(9) Å are significantly shorter than the Sn–K distances of low-valent Sn(II) and Sn(I) complexes containing a K^+^ counterion, which report values within the range 3.460(4)–3.7202(1) Å.^69−72^ The short Sn–K distances in **2** indicates a strong interaction between the two atoms, though whether this arises from the rigid sterics or electron-withdrawing influence of the **bc** ligands cannot be determined. The counteranion charge should be delocalized over the biscarborane cages.^1,3^

**Figure 4:**
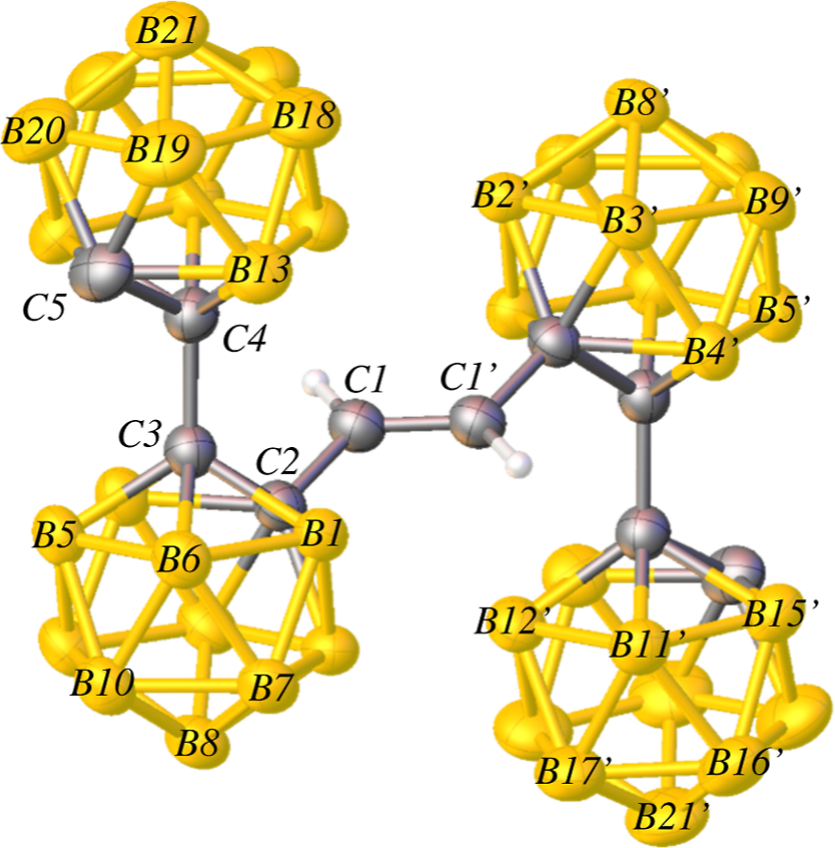
Thermal ellipsoid plot (50%) of **3**. Cage-bonded H atoms are not shown for clarity. Selected bond lengths (Å) and angles (deg): C1–C1′ = 1.319(4), C1–C2 = 1.488(3), C3–C4 = 1.533(3), C2–C1–C1′ = 123.1(2).

Structural data for compound **3** shows an inversion center which imposes a *trans* configuration around the central C1–C1′ bond (Figure 4). The C1–C1′ bond distance (1.319(4) Å) and C2–C1–C1′ bond angle (123.1(2)°) are consistent with the presence of a C=C double bond.^67^ Overall, compound **3** has *C*_2*h*_ symmetry. A series of dicarboranyl ethenes R(C_2_B_10_H_10_)CH=CH(C_2_B_10_H_10_)R (R = Ph or C_6_H_4_Me-*p*) analogous to compound **3** similarly contain a trans C=C double bond.^46^ More recently, carborane clusters linked via a phenyl group have also been reported, generally containing the formula (C_2_B_10_H_11_)–Ph–(C_2_B_10_H_11_).^73−75^ To the best of our knowledge, compound **3** is the first dibiscarboranyl ethene in the literature.

### Spectroscopy

Compounds **1-3** were characterized by ^1^H NMR, ^11^B NMR, ^13^C NMR, UV–vis, and IR spectroscopy. Compound **1** was also characterized by ^119^Sn NMR spectroscopy.

The ^1^H NMR spectrum for **1** displays the coordinated THF proton signals at 1.40 and 3.55 ppm, which is in the same range as those of other THF-coordinated Sn(II) complexes (δ_CH_2_(3,4)_ = 1.3–1.8; δ_CH_2_(2,5)_ = 3.5–3.7)^65−68^ as well as signals due to free THF in C_6_D_6_ (δ_CH_2_(3,4)_ = 1.43; δ_CH_2_(2,5)_ = 3.57).^43^

The ^119^Sn NMR spectrum for **1** displays a signal at −137.31 ppm. Related (**bc**)Sn compounds have ^119^Sn signals further downfield than compound **1**, with (**bc**)SnMe_2_ having a signal at −21.22 ppm in d^8^-THF and the methyl-substituted derivative (**Mebc**)SnMe_2_ (**Mebc** = 8,8′,9,9′,10,10′,12,12′-octamethyl-1,1′-bis(*o*-carborane)) at 9.20 ppm in d^8^-THF and 53.10 ppm in C_6_D_6_ (Table 2).^20^ A decrease in the coordination environment around the Sn atom usually results in a downfield shift of the ^119^Sn resonance.^76^ Nonetheless, 3-coordinate **1** displays an upfield shift in comparison to the 4-coordinate (**bc**)SnMe_2_ and (**Mebc**)SnMe_2_. The three-coordinate, THF-bonded complexes Sn[OC(C_4_H_3_S)_3_]_2_(THF)^65^ and [Sn(box)(THF)]^+^ (box = 1,1-bis[(4*S*)-4-phenyl-1,3-oxazolin-2-yl]ethane)^67^ report ^119^Sn NMR signals upfield of the chemical shifts of **1** at −244.5 and −377.1 ppm, respectively. As the signal for **1** is observed between its tetra-coordinated analogues and Sn(II) ← THF derivatives, THF coordination aids in shielding the tin atom, leading to a more shielded Sn atom than that in (**bc**)SnMe_2_ and (**Mebc**)SnMe_2_, while the electron-withdrawing effect of the **bc** ligand causes a deshielding on Sn relative to other Sn(II) ← THF complexes.

**Table 2:**
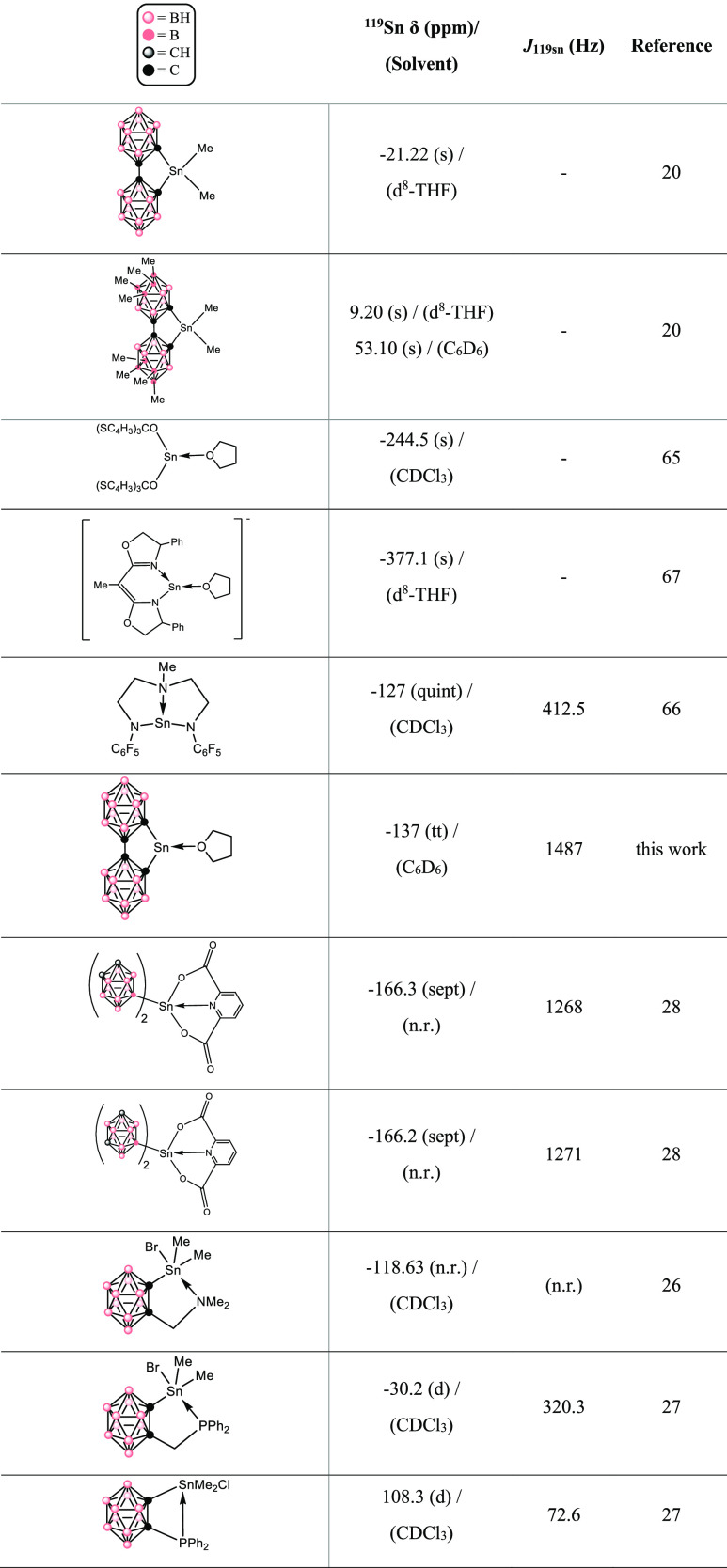
^119^Sn NMR Chemical Shifts for **1** and Selected Compounds^a^

a n.r.: not reported.

**Figure 5:**
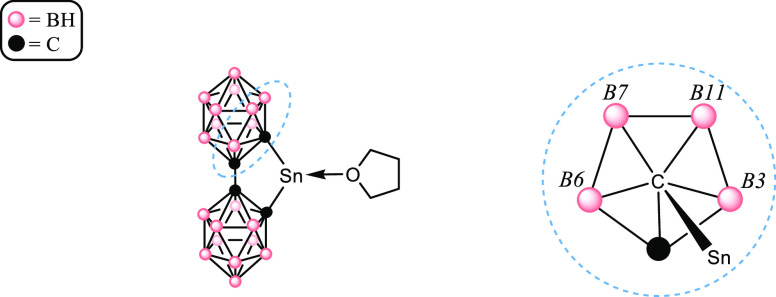
Left: Tin-bonded carbon vertex face is marked with a blue circle. Right: “Front” view of the blue-circled face, showing the two chemical environments of B3/B6 (italicized) vs B7/B11.

The triplet of triplets which occurs in the ^119^Sn NMR spectrum of compound **1** is unusual, given the absence of a Sn–H signal in its ^1^H NMR and IR spectra and X-ray structural data. Additionally, ^119^Sn NMR signals for Sn(II) ← THF complexes often appear as singlets in the spectrum (Table 2).^65−67^ However, multivalent Sn complexes bonded to electron-withdrawing groups and supported by a Sn ← X (X = N or P) dative bond report multiplets in their ^119^Sn NMR spectra (Table 2).^26−28,66^ The carboranyl–tin complexes by Gielen and coworkers report 1:2:3:4:3:2:1 septets in their corresponding ^119^Sn NMR spectra at −166.3 and −166.2 ppm, with coupling constants of 1268 and 1271 Hz^28^ similar to the coupling constant for the ^119^Sn NMR signal of **1** (1487 Hz). In addition, carboranyl tin complexes supported by a Sn ← X dative bond (X = N or P) typically observe doublets in the ^119^Sn NMR spectrum, depending on both the identity of the X atom and coordination about Sn.^26−28,30,76−78^ The splitting patterns which appear in the ^119^Sn NMR spectra of compound **1** and carboranyl tin coordination complexes presumably arise from long-range nuclear spin–spin coupling between the carboranyl boron and tin nuclei.^79,80^ The quadrupolar relaxation rate of the ^11^B nucleus (*I* = 3/2) is known to influence the appearance of the resonances of nuclei with spin *I* = 1/2, such as ^119^Sn.^79−81^ Specific to compound **1**, the four boron atoms bonded to the tin-bound carbon atom (B3, B6, B7, and B11) exist in two different chemical environments due to the *C*_2*v*_ symmetry of the *o*-carborane cage (Figure 5), likely causing the triplet of triplets displayed in the ^119^Sn spectrum of **1**.

Despite numerous attempts to record spectra, with the use of a wide variety of parameters, the ^119^Sn NMR signal for compound **2** could not be detected. Problems in obtaining the ^119^Sn data were also encountered for the dianions K_2_[Ar^iPr^^6^SnSnAr^iPr^^6^], which was hypothesized to be caused by the unsymmetric electron environment at the Sn atoms, which may cause rapid relaxation through the high anisotropy of the chemical shift tensor.^70,82^ The THF ligand in **1** appears to stabilize the electron environment at the Sn atom to facilitate detection of a signal. In addition, though the ^11^B NMR spectra of **1** and **2** are both proton-decoupled, the spectrum for **2** displays tin satellites at −14 and −5 ppm (See Supporting Information, Figure S5) that are absent in the spectrum for **1**. This difference can also be attributed to the coordination of THF to the ^119^Sn nucleus in **1** but not **2**.

The UV–vis spectrum of **1** displays two absorptions in the near-UV region at 280 and 345 nm. These absorptions persist in **2**, appearing also at 280 and 345 nm regardless of whether a THF or K ion is coordinated to Sn. The similar absorptions in the UV–vis spectra for **1** and **2** suggests that compounds **1** and **2** exist as the same compound in the solution phase. The relatively intense absorptions at 280 nm and similarly at 284 nm in the UV–vis spectrum for **3** can be tentatively assigned to an energy transfer on the bis-carborane ligand. The near-UV vis region of the absorption bands of **1** and **2** suggests a high-energy HOMO → LUMO transition of the (**bc**)Sn compounds.

Compound **3** exhibits spectroscopic features characteristic of alkenes. The olefin protons appear at 5.43 and 6.10 ppm in the ^1^H NMR spectrum and the olefin carbon at 2.65 ppm in the ^13^C NMR spectrum at the high frequency shifts indicative of more conjugated alkenes.^83^ The UV–vis spectrum of **3** displays a shoulder at 334 nm, corresponding to an olefin π → π* transition at a relatively longer wavelength for alkenes groups, further confirming a conjugated alkene.^83^ Interestingly, a ν_C=C_ stretching frequency in the IR spectrum within the characteristic 1680–1640 cm^–1^ region is not observed.

## Conclusions

The syntheses for **1–3** proceeded in a similar way to each other with only simple modifications in solvents or reaction time. In THF solvent, the synthetic procedure gave the THF-coordinated **1**, while using a stepwise benzene and dichloromethane solvent mixture gave **2**. Shortening the reaction period of the step that generates the dipotassium salt from 24–48 h to 9–12 h gave the alkene **3**. Compound **1** exists as a Lewis acid–base pair with THF, as displayed in the X-ray structural data. Furthermore, the **bc** ligand platform confers interesting spectroscopic characteristics in the ^119^Sn NMR spectrum that is unusual for Sn(II)–THF complexes but usual for organotin complexes featuring electron-withdrawing ligands like carboranes. X-ray structural data for **2** show that the Sn atom contains a similar structural motif to that of **1**. Compound **3** is the first example of a dibiscarborane-supported alkene.

## Supporting Information Available

Crystallographic data and NMR, IR, and UV–vis spectra (PDF)
