# Correction to “Synthesis, Structure, and Spectroscopy of the Biscarboranyl Stannylenes (bc)Sn·THF and K_2_[(bc)Sn]_2_ (bc = 1,1′(*ortho*-Biscarborane)) and Dibiscarboranyl Ethene (bc)CH=CH(bc)”

**DOI:** 10.1021/acs.organomet.3c00319

**Published:** 2023-08-10

**Authors:** Alice C. Phung, James C. Fettinger, Philip P. Power

In the original title publication the compound **2** was reported to have the formula K_2_[(**bc**)Sn]_2_ (**bc**=1,1′-bis(*ortho*-biscarborane)). But a re-examination and rerefinement of the X-ray crystallographic data for **2** indicate that the correct formula is [(**bc**)Sn]_2_KCl. As reported earlier, one of the K^+^ ions acts as a countercation which is coordinated to the B–H vertices of the **bc** cage, but the second K^+^ ion, which was said to bridge the two tin atoms in the original report, has been reformulated as a Cl^–^ ion, thereby forming a Sn–Cl–Sn bridging unit between two (**bc**)Sn moieties, as in the {(**bc**)Sn-μ-Cl-Sn(**bc**)}^−^ anion, as well as lowering the *R* value from 2.43% to 2.23%. Organotin complexes containing a Sn–Cl–Sn moiety typically have bridging Sn–Cl distances in the range 2.540(2)–2.967(1) Å,^2−11^ and this fragment of compound **2** features Sn–Cl bond distances of 2.5868(8) and 2.5874(8) Å, which are at the lower end of the above range. The relatively short Sn–Cl distances in **2** indicate a strong interaction between the two ions, but whether this is a consequence of the rigid structure or electron-withdrawing influence of the **bc** ligand is not apparent. The drawings in Figure 1 below illustrate the corrected X-ray crystal formula for compound **2**, and Figure 1c in particular clearly illustrates the bridging atom as Cl^–^ seems more chemically sensible than when the species is formulated with a bridging K^+^ cation.

**Figure 1:**
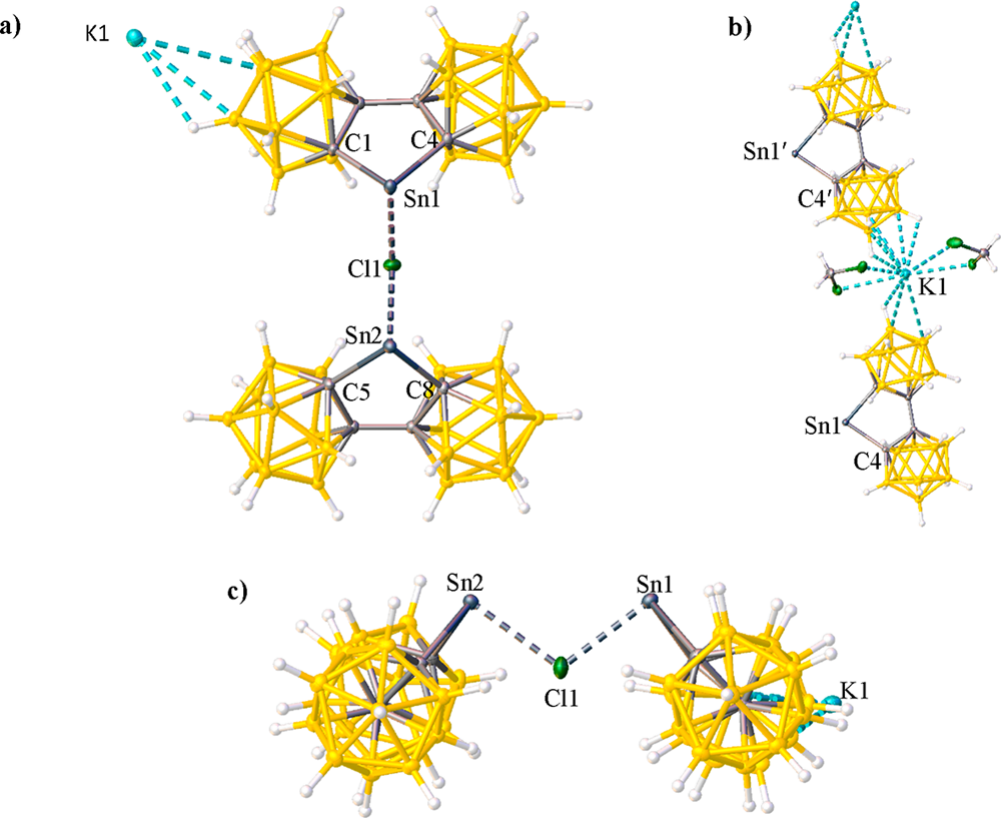
Thermal ellipsoid plot (50%) of **2**. (a) View of **2** to show coordination positions of K1 and Cl1. The CH_2_Cl_2_ solvent molecules are not shown for clarity. (b) Expanded view of **2** to show coordination of K1. (c) View of **2** to show coordination of Cl1. CH_2_Cl_2_ solvent molecules are not shown for clarity. Selected bond lengths (Å) and angles (deg): C1–Sn1 = 2.276(3), C4–Sn1 = 2.309(3), C5–Sn2 = 2.288(4), C8–Sn2 = 2.289(3), Cl1–Sn1 = 2.5868(8), Cl1–Sn2 = 2.5874(8), C1–Sn1–C4 = 81.69(11), C5–Sn2–C8 = 81.86(12), Sn1–Cl1–Sn2 = 106.98(3), C1–Sn1–Cl1 = 88.27(7), C4–Sn1–Cl1 = 94.11(7), C5–Sn2–Cl1 = 92.62(7), C8–Sn2–Cl1 = 90.91(7).
