# Supplementary material for: Synthesis, Structure, and Spectroscopy of the Biscarboranyl Stannylenes (bc)Sn·THF and K2[(bc)Sn]2 (bc = 1,1′(ortho-Biscarborane)) and Dibiscarboranyl Ethene (bc)CH=CH(bc)
Source: Organometallics. 2023 Jun 26;42(13):1649–57. doi: 10.1021/acs.organomet.3c00190 (PMC10337257; doi:10.1021/acs.organomet.3c00190)
Supplement: Supplementary file 1 — om3c00190_si_001.pdf [file om3c00190_si_001.pdf]

Electronic Supplementary Information

Synthesis, Structure, Spectroscopy of the Biscarboranyl Stannylenes $K_2[(bc)Sn]_2$ and (bc)Sn·THF (bc = 1,1′-biscarborane) and Dibiscarboranyl Ethene (bc)HC=CH(bc)

Alice C. Phung,[1] James C. Fettinger,[1] Philip P. Power[1]*

[1]Department of Chemistry, University of California, 1 Shields Avenue, Davis, California 95616

Table of Contents

X-ray crystallography
Table S1. Crystallographic and Data Collection Parameters for **1-3** S2

NMR spectra
Figure S1. ^{1}H NMR spectrum of **1** in C_6D_6 at 298K. S3
Figure S2. ^{11}B{^{1}H} NMR spectrum of **1** in C_6D_6 at 298K. S3
Figure S3. ^{13}C{^{1}H} NMR spectrum of **1** in C_6D_6 at 298K. S4
Figure S4. ^{1}H NMR spectrum of **2** in C_6D_6 at 298K. S4
Figure S5. ^{11}B{^{1}H} NMR spectrum of **2** in C_6D_6 at 298K. S5
Figure S6. ^{13}C{^{1}H} NMR spectrum of **2** in C_6D_6 at 298K. S5
Figure S7. ^{119}Sn NMR spectrum of **2** in C6D6 at 298K. S6
Figure S8. ^{1}H NMR spectrum of **3** in C_6D_6 at 298K. S6
Figure S9. ^{11}B{^{1}H} NMR spectrum of **3** in C_6D_6 at 298K. S7
Figure S10. ^{13}C{^{1}H} NMR spectrum of **3** in C_6D_6 at 298K. S7

UV-vis spectra
Figure S11. UV-vis spectrum of **1** in toluene at 298K. S8
Figure S12. UV-vis spectrum of **2** in toluene at 298K. S8
Figure S13. UV-vis spectra of **3** in toluene at 298K. S9

IR spectra
Figure S14. Infrared spectrum of **1**. S10
Figure S15. Infrared spectrum of **2**. S11
Figure S16. Infrared spectrum of **3**. S12

References S12

X-ray Crystallography

Crystals of **1**, **2**, and **3** were removed from a Schlenk flask under a stream of nitrogen and immediately covered with a layer of hydrocarbon oil. A suitable crystal was selected, attached to a glass fiber on a copper pin and quickly placed in the cold N_2 stream on the diffractometer. Data was collected at 100 K on a Bruker APEX DUO diffractometer with Mo Kα radiation (λ = 0.71073 Å). Absorption corrections were applied using SADABS.[S1] The crystal structures were solved by direct methods and refined by full matrix least-squares procedures in SHELXTL.[S2] All non-H atoms were refined anisotropically.

Table S1. Selected X-ray Crystallographic data for **1-3**.

	**1**	**2**	**3**
**formula**	$C_{17}H_{58}B_{40}Cl_2O_2Sn_2$	$C_{10}H_{44}B_{40}Cl_4K_2Sn_2$	$C_{16}H_{50}B_{40}$
**fw**	1035.31	1054.23	674.96
**color, habit**	Colorless plate	Colorless shard	Yellow plate
**space group**	$P\bar{1}$	$P2_1$	*C2/c*
***a*, Å**	11.86820(10)	12.7653(3)	31.509(4)
***b*, Å**	13.8376(2)	12.7550(3)	12.6857(17)
***c*, Å**	14.7426(2)	14.8445(4)	10.2461(13)
***α*, °**	101.2593(7)	90	90
***β*, °**	98.6642(6)	114.5093(11)	94.241(3)
***γ*, °**	92.6166(8)	90	90
***V*, Å^3**	2340.40(5)	2199.22(10)	4084.3(9)
Z	2	2	4
**crystal size, mm^3**	0.219 x 0.179 x 0.080	0.464 x 0.401 x 0.280	0.410 x 0.239 x 0.118
**d_{calc}, Mg cm^{-3}**	1.469	1.592	1.098
**abs. μ, mm^{-1}**	9.700	1.586	0.047
***2θ*, °**	3.098 to 69.471	1.753 to 30.753	2.593 to 25.249
**R(int)**	0.0330	R1 = 0.0243	0.0569
**obs. reflns. [I>2σ(I)]**	6881	13126	2642
**data/restraints/parameters**	8069 / 22 / 595	13561 / 14 / 695	3705 / 14 / 369
**R_1, obsd. reflns.**	0.0411	0.0255	0.0803

NMR Spectra

Figure S1. ^{1}H NMR spectrum of **1** in C_6D_6 at 298K.

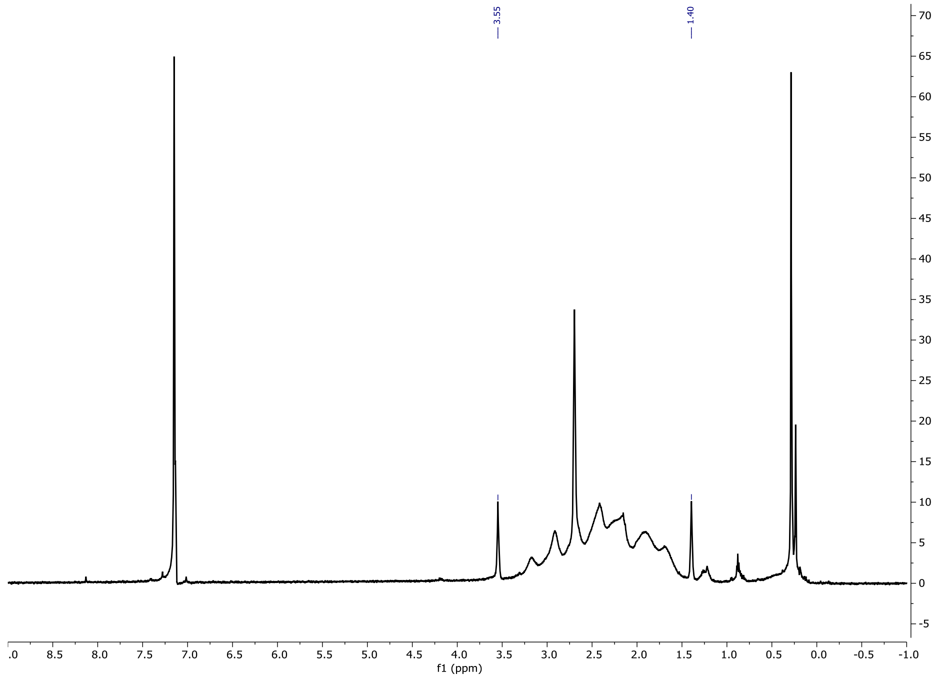

Note: δ 0.29: residual grease. δ 0.89, 1.24: residual hexanes. δ 7.15: residual benzene.

Figure S2. $^{11}B\{^{1}H\}$ NMR spectrum of **1** in C_6D_6 at 298K.

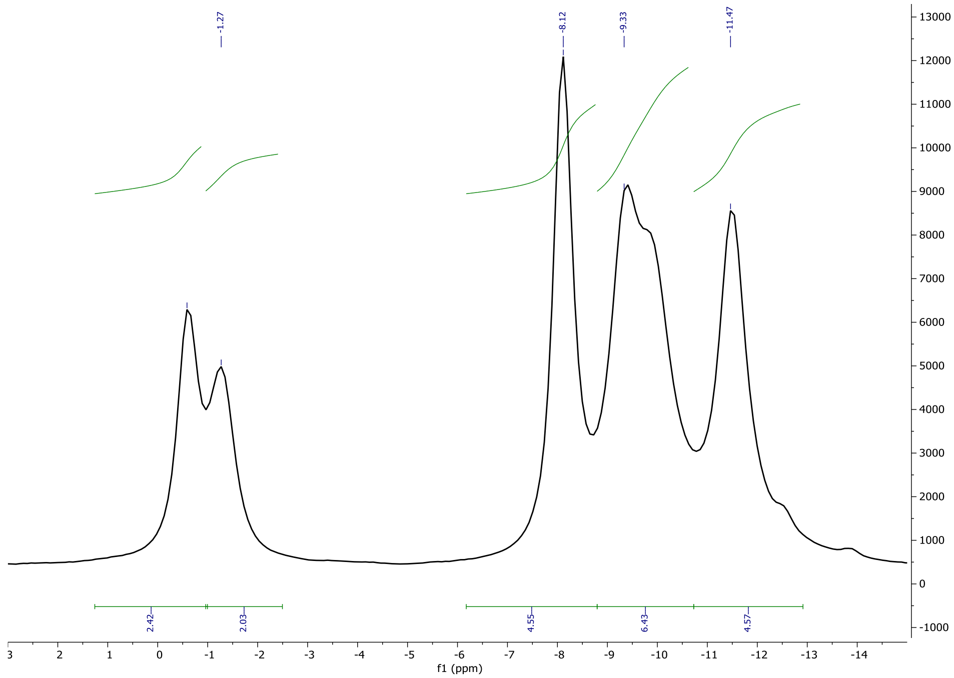

NMR Spectra

Figure S3. $^{13}C\{^{1}H\}$ NMR spectrum of **1** in C_6D_6 at 298K.

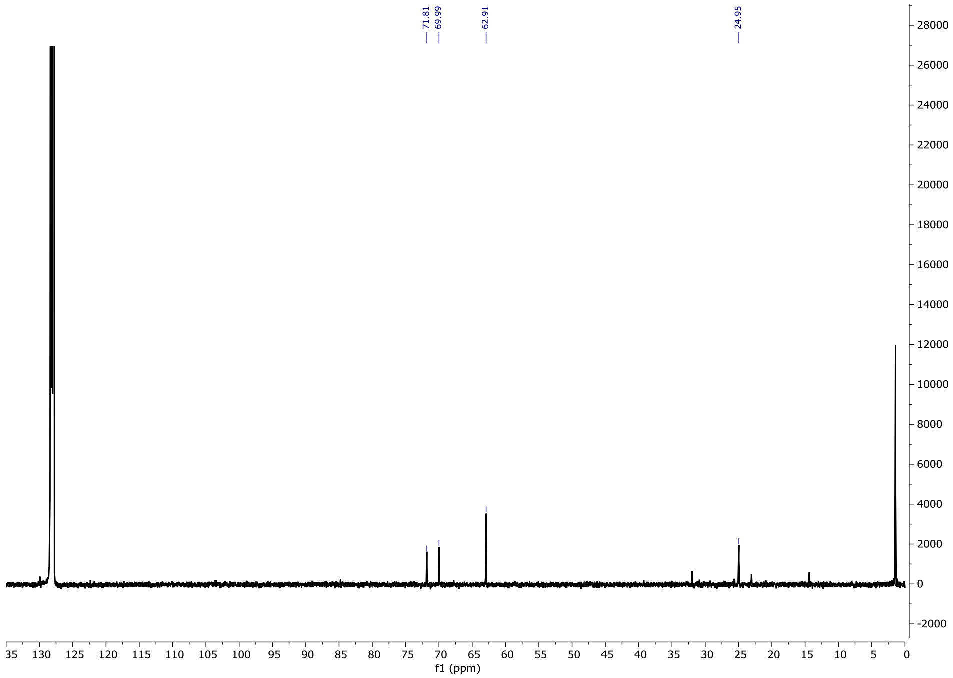

Note: δ 1.42: residual grease. δ 14.37, 23.06, 31.97: residual hexanes. δ 128.06: residual benzene.

Figure S4. ^{1}H NMR spectrum of **2** in C_6D_6 at 298K.

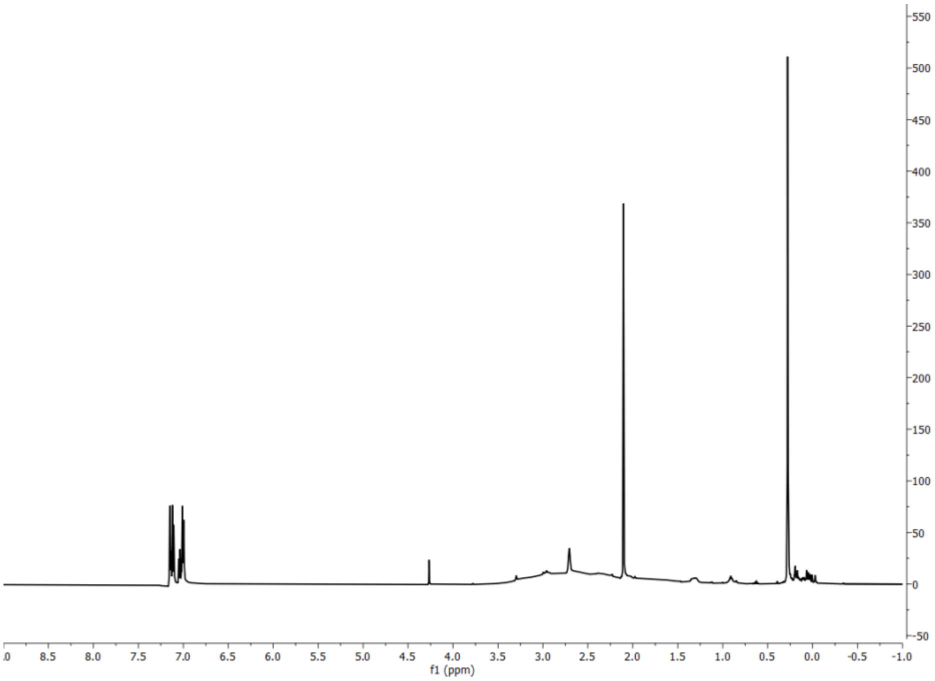

Note: δ 0.29: residual grease. δ 2.10, 7.0 – 7.2: residual toluene. δ 4.27: residual dichloromethane.

NMR Spectra

Figure S5. $^{11}B\{^{1}H\}$ NMR spectrum of **2** in C_6D_6 at 298K

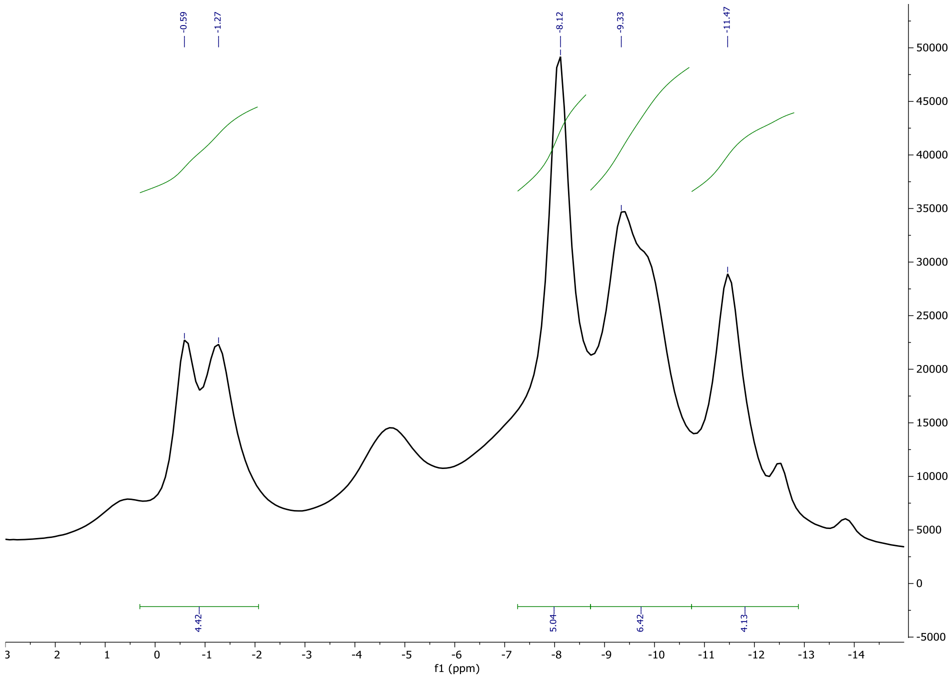

Figure S6. $^{13}C\{^{1}H\}$ NMR spectrum of **2** in C_6D_6 at 298K.

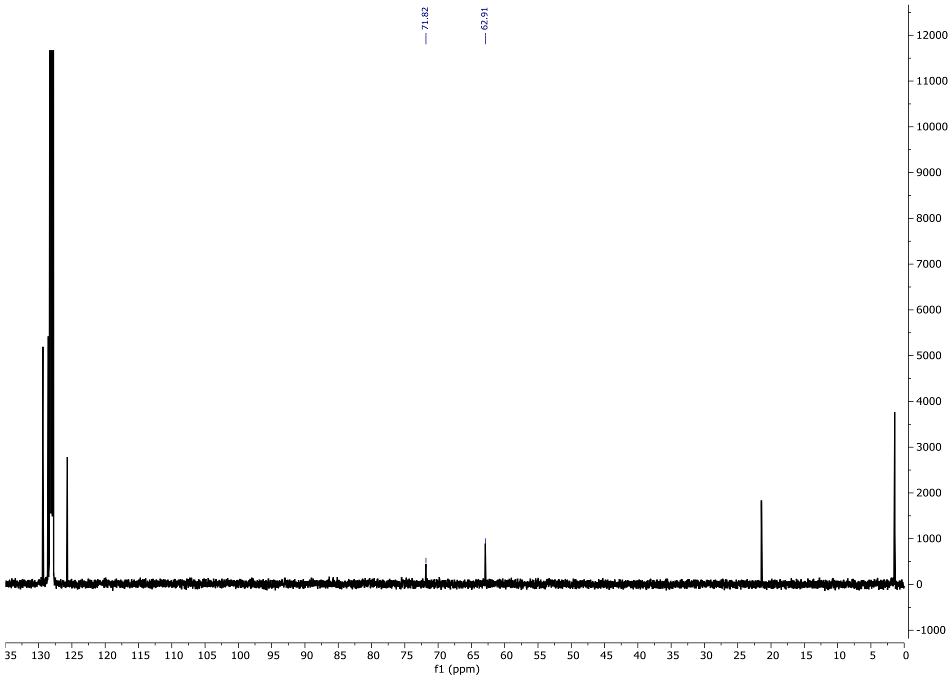

Note: *δ* 1.42: residual grease. *δ* 21.98, 126.23, 129.88: residual toluene.

NMR Spectra

Figure S7. $^{119}Sn\{^1H\}$ NMR spectrum of **2** in C_6D_6 at 298K.

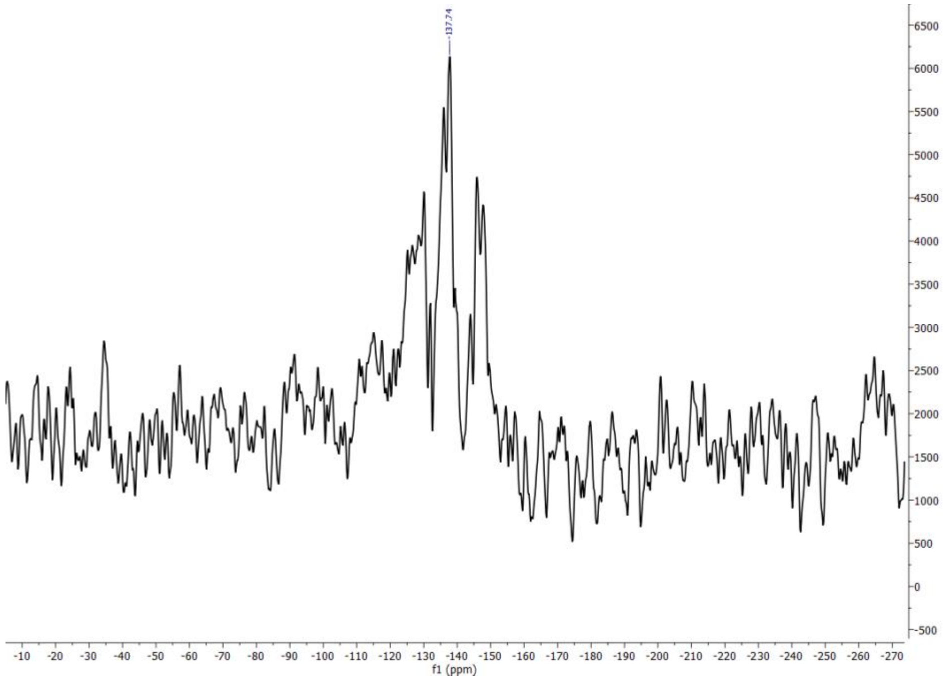

Figure S8. ^1H NMR spectrum of **3** in C_6D_6 at 298K.

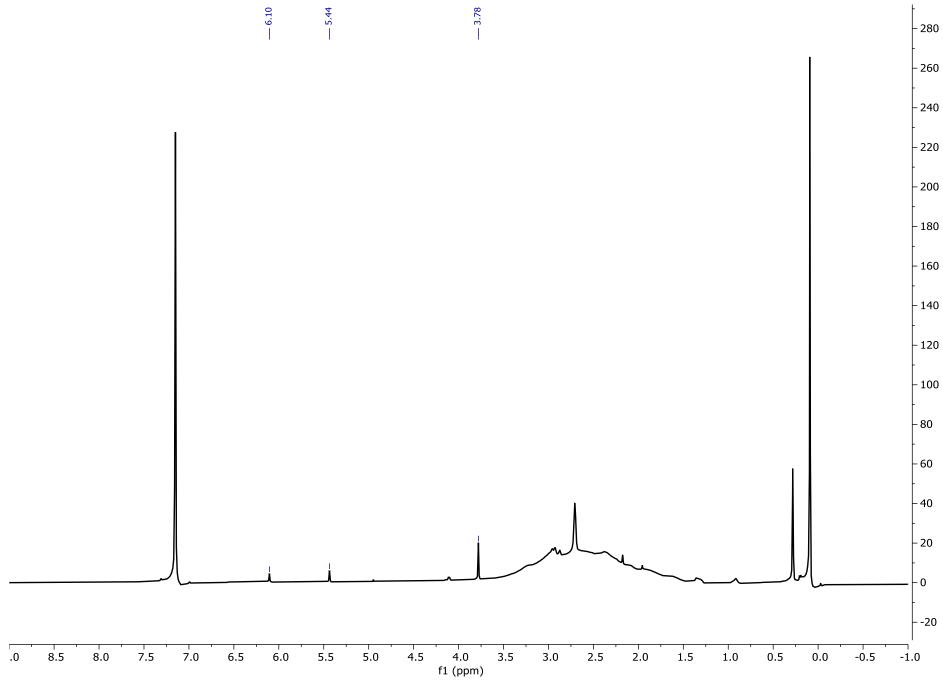

Note: δ 0.09, 0.29: residual grease. δ 0.89, 1.24: residual hexanes. δ 7.15: residual benzene.

NMR Spectra

Figure S9. $^{11}B\{^1H\}$ NMR spectrum of **3** in C_6D_6 at 298K.

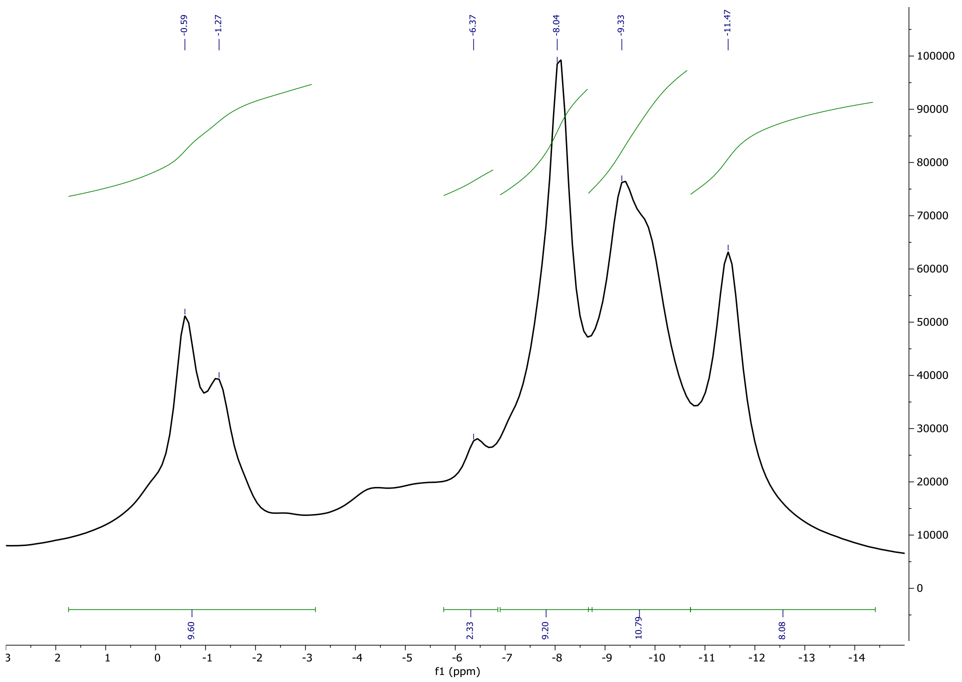

Figure S10. $^{13}C\{^1H\}$ NMR spectrum of **3** in C_6D_6 at 298K

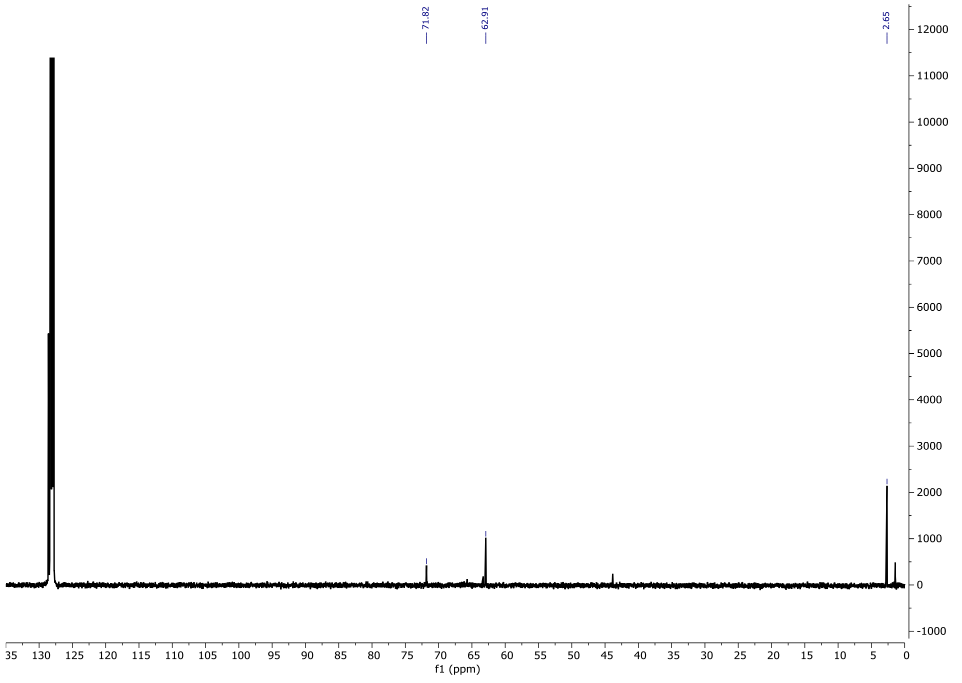

Note: δ 1.42: residual grease. δ 128.06: residual benzene.

UV-vis Spectra

Figure S11. UV-vis spectrum of **1** in toluene at 298K.

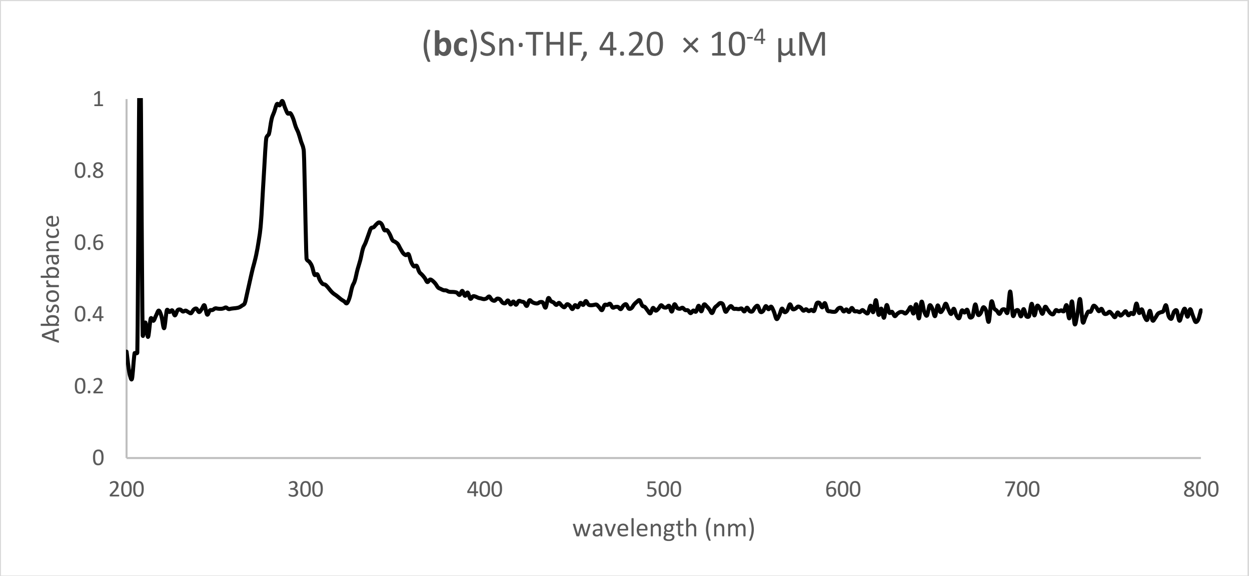

Figure S12. UV-vis spectrum of **2** in toluene at 298K.

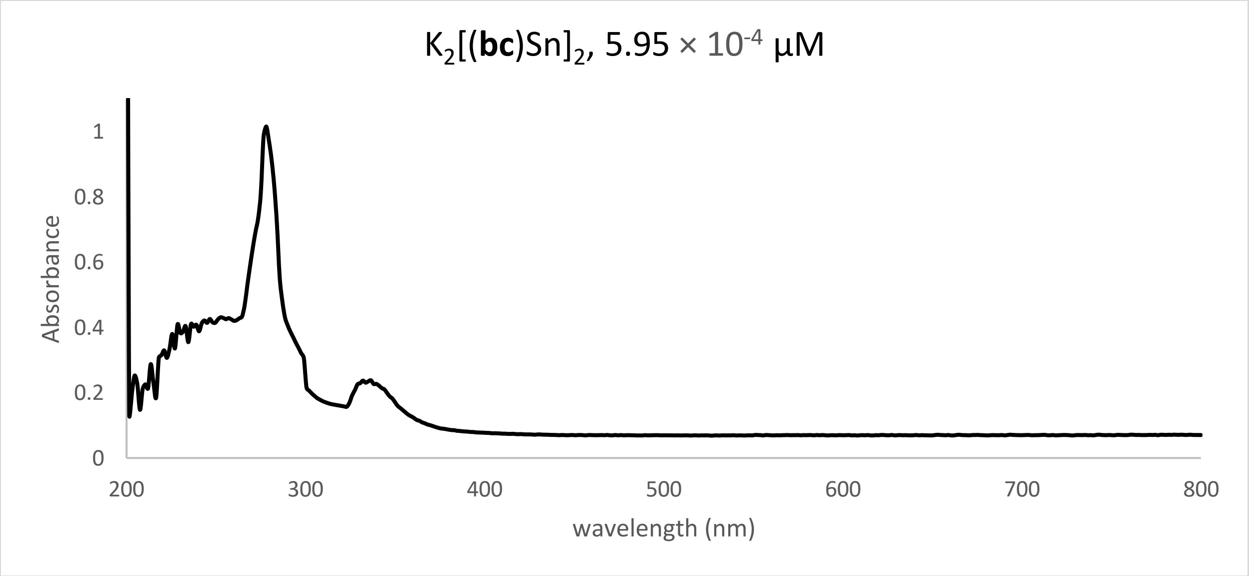

UV-vis Spectra

Figure S13. UV-vis spectra of **3** in toluene at 298K.

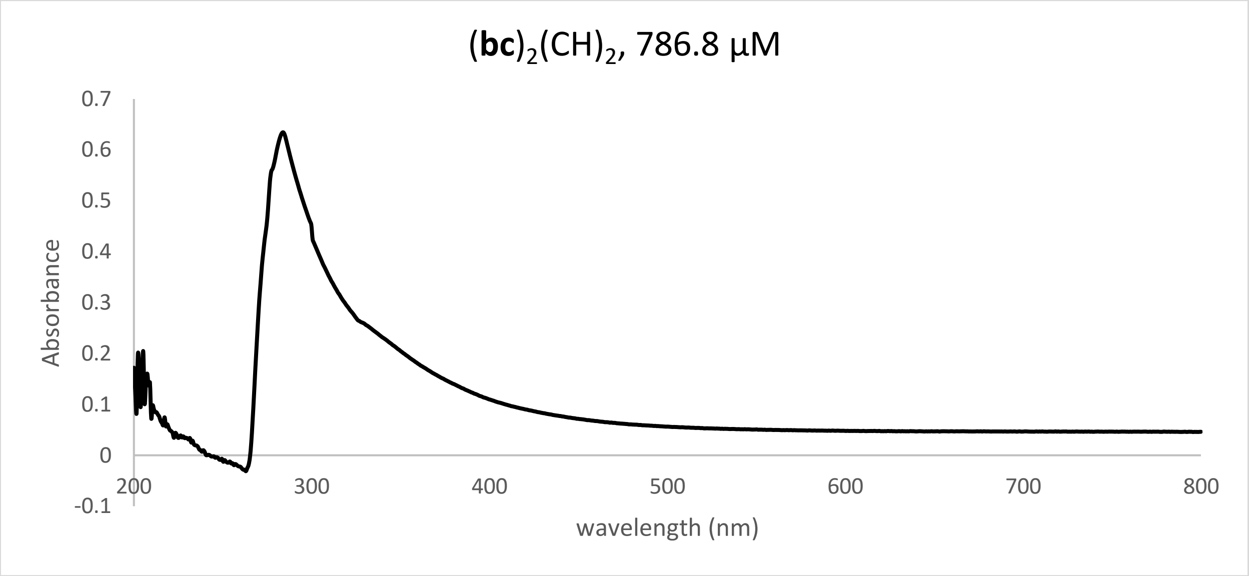

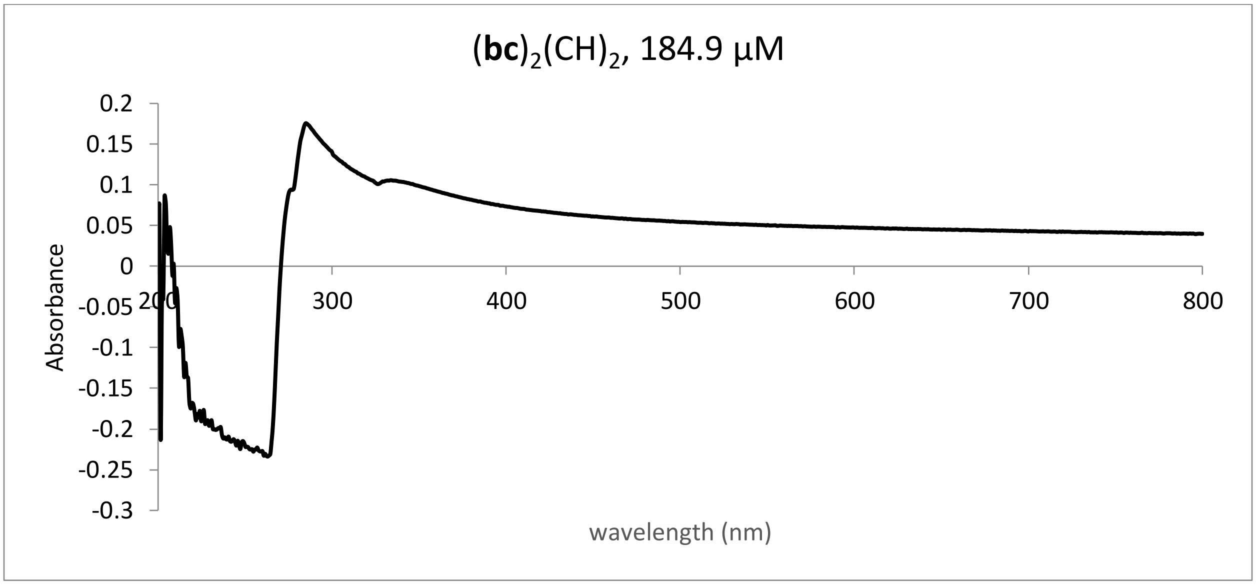

Note: A more dilute spectrum (bottom) is provided to clearly display the shoulder at 334 nm.

IR spectra

Figure S14. Infrared spectrum of **1**.

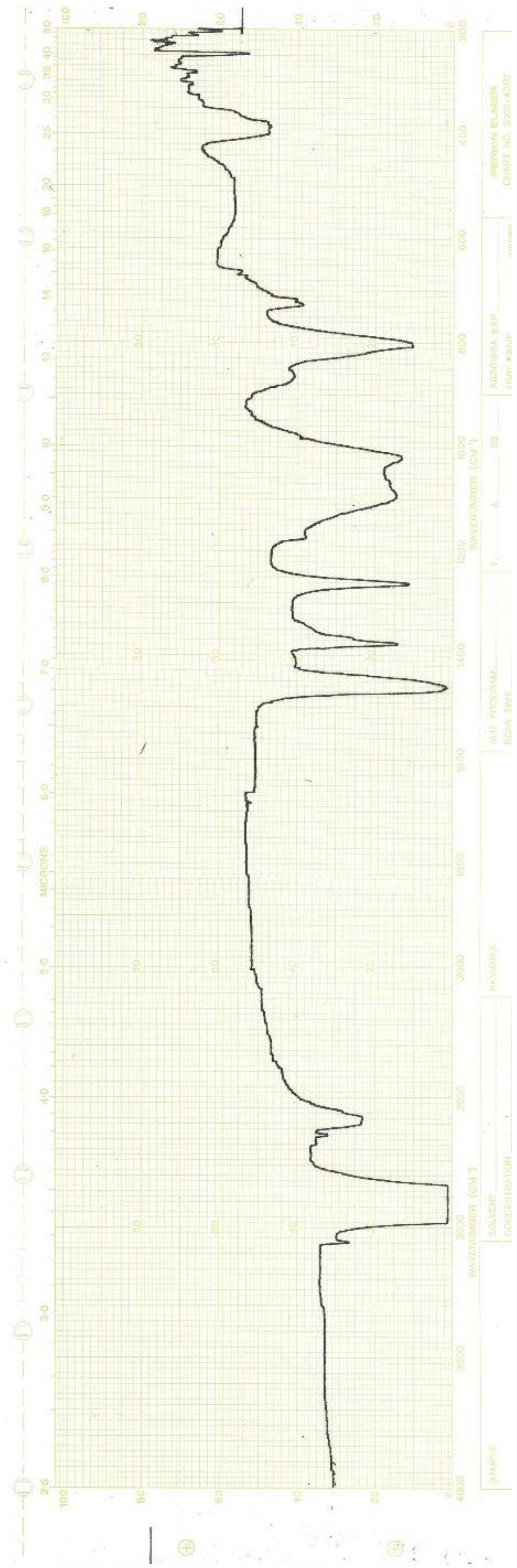

IR spectra

Figure S15. Infrared spectrum of **2**.

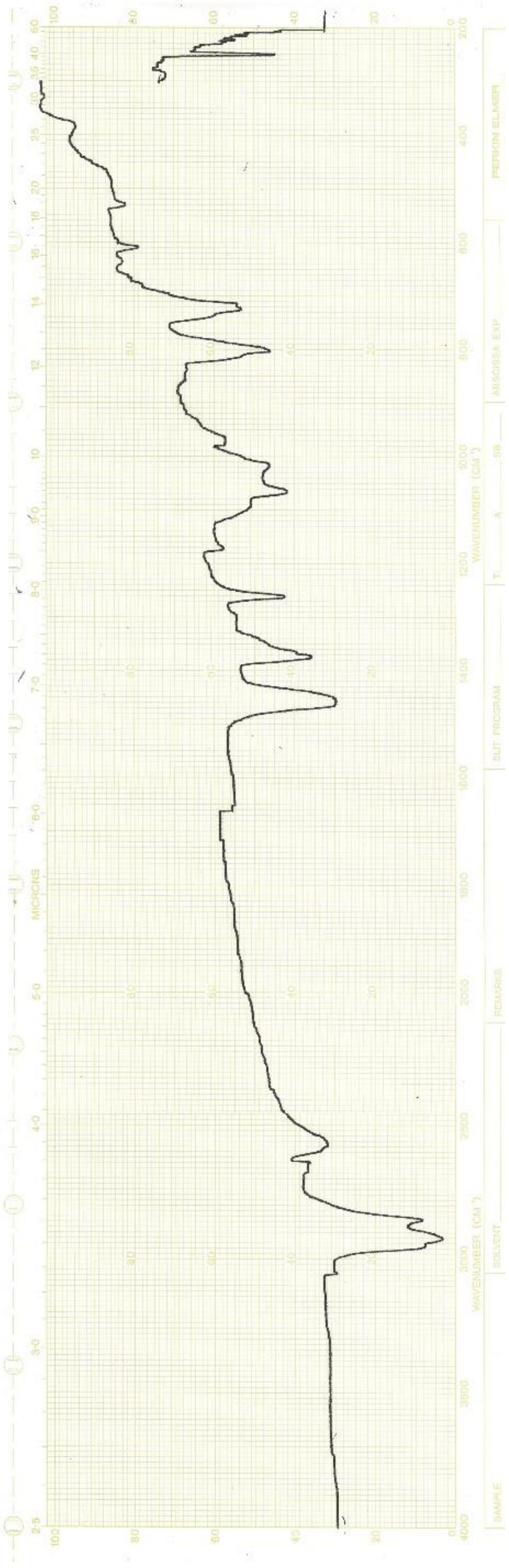

IR spectra

Figure S16. Infrared spectrum of **3**.

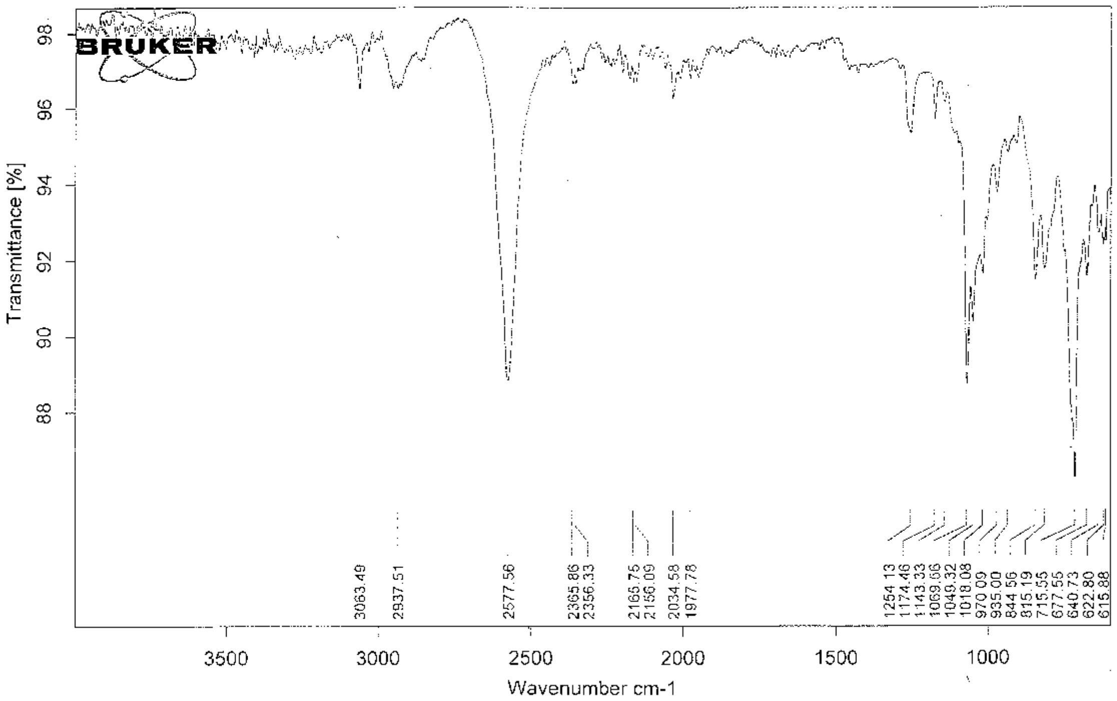

References

S1 Bruker, Bruker AXS Inc., Madison, Wisconsin, USA, **2001**
S2 G. M. Sheldrick, Acta Cryst. A 2015, 71, 3-8.
S3 Dolomanov, O. V.; Bourhis, L. J.; Gildea, R. J.; Howard, J. A. K.; Puschmann, H.; *OLEX2*: A complete structure solution, refinement and analysis program. J. Appl. Cryst, **2009**, *42*, 339-341.
